# An Adaptive Sliding-Mode Iterative Constant-force Control Method for Robotic Belt Grinding Based on a One-Dimensional Force Sensor

**DOI:** 10.3390/s19071635

**Published:** 2019-04-05

**Authors:** Tie Zhang, Ye Yu, Yanbiao Zou

**Affiliations:** School of Mechanical and Automotive Engineering, South China University of Technology, Guangzhou 510000, China; 201720101200@mail.scut.edu.cn (Y.Y.); ybzou@scut.edu.cn (Y.Z.)

**Keywords:** robot, abrasive belt grinding, constant-force control, adaptive sliding-mode control, iterative learning

## Abstract

To improve the processing quality and efficiency of robotic belt grinding, an adaptive sliding-mode iterative constant-force control method for a 6-DOF robotic belt grinding platform is proposed based on a one-dimension force sensor. In the investigation, first, the relationship between the normal and the tangential forces of the grinding contact force is revealed, and a simplified grinding force mapping relationship is presented for the application to one-dimension force sensors. Next, the relationship between the deformation and the grinding depth during the grinding is discussed, and a deformation-based dynamic model describing robotic belt grinding is established. Then, aiming at an application scene of robot belt grinding, an adaptive iterative learning method is put forward, which is combined with sliding mode control to overcome the uncertainty of the grinding force and improve the stability of the control system. Finally, some experiments were carried out and the results show that, after ten times iterations, the grinding force fluctuation becomes less than 2N, the mean value, standard deviation and variance of the grinding force error’s absolute value all significantly decrease, and that the surface quality of the machined parts significantly improves. All these demonstrate that the proposed force control method is effective and that the proposed algorithm is fast in convergence and strong in adaptability.

## 1. Introduction

As a finishing process, abrasive belt grinding not only achieves high material removal rates, but also can be used to improve the surface roughness of components [1]. By integrating a multi-degree industrial robot as a manipulator, a flexible manufacturing cell can be formed, which is especially suitable for processing surfaces with complicated geometries, such as turbine blades or faucets [2]. It can avoids a series of problems caused by manual grinding and CNC grinding, such as the health problems caused by the harsh processing environment, low processing efficiency, increasing labor costs [3], poor stability, and insufficient consistency [4]. Therefore, there have been many studies on robotic belt grinding, and some of them have addressed the problems of robotic offline programming [5,6] and robotic trajectory planning [7,8,9]. These methods can improve the machining quality of workpieces to some extent. Some related studies have shown that by controlling the required grinding force, the material removal rate can be indirectly controlled to improve the processing quality of the workpieces, and the phenomenon of over- and under-cutting of the workpieces caused by an improper contact force can thereby be avoided [10]. These findings indicate that controlling the normal contact force is also the key to improving the grinding quality of abrasive belt. Thus, an increasing number of researchers are attempting to achieve the desired force control.

To achieve robotic grinding force control, some researchers have adopted passive force control methods. Li et al. [11] developed an auxiliary pneumatic flexible flange that is mounted at the end of the industrial robots for the robotic grinding process. Qi et al. [12] proposed the concept of the working accuracy for an industrial robot application system. On this basis, the working accuracy model of the grinding process was derived, and a robot operation error measurement and compensation system was designed to improve the overall performance of an industrial robot belt grinding system. In addition, Wang et al. [13] analyzed and optimized the dexterity of the robotic belt grinding system. The grinding quality has been effectively improved through these great studies on the working accuracy of robotic belt grinding and the force compensation during the grinding, but the dynamic range of the force response and the accuracy of the robotic end position in the grinding process have been reduced. 

To overcome these shortcomings of passive force control, active force control [14] came into being and has become a major direction in the field of robot research. Currently, the research on robotic active force control can be essentially divided into two categories: the force control based on traditional strategies and the force control based on intelligent strategies. The traditional control methods can be further generally divided into force/position hybrid control [15] and impedance control [16]. Zhang et al. [17] applied force/position hybrid control to a speed servo-based grinding robot, and controlled the grinding force by adjusting the rotational speed of the robot’s joints. Lahr et al. [18] also studied the force/position hybrid control of robot grinding force and discussed its feasibility in ceramic cutting. As for impedance control, Zhang et al. [19] proposed a hybrid passive/active force control scheme for a grinding tap with an industrial robot for a series of outstanding problems in the grinding industry. He designed an abrasive belt grinder with a dynamic position adjustment as the passive force control device and then used impedance control for the active force control to achieve the control effect. On the other hand, Lu et al. [20] proposed a sliding-mode-based impedance controller with a continuous PI control near the surface of the sliding mode to avoid chatter and reduce steady-state errors. Although these traditional force control strategies can achieve certain control effects, due to the non-linearity and a large amount of uncertainty in robotic belt grinding, it is difficult to achieve satisfactory results using these control methods.

Taking these problems into account, some scholars have proposed some intelligent control methods that can effectively identify nonlinear systems. For example, Song et al. [21] proposed an intelligent control method that can calculate the optimal control parameters in real time, but the experiment also requires a certain amount of training to achieve the control effect. Seraji et al. [22] proposed adaptive impedance control, which has a better force tracking effect under the condition of unknown environmental parameters through direct and indirect methods, but too many of the adaptive gain parameters cannot be practically adjusted. To overcome this problem, Jung et al. [23] designed an adaptive control law based on the error obtained by the force feedback, so that only a simple adaptive gain is needed to achieve a good control effect. However, it is difficult to obtain this appropriate gain. Chan et al. [24] presented a data-efficient learning variable impedance control method which improves the flexibility and adaptability of the system. 

The grinding effect of the robot belt is finally reflected in the force control effect and the surface roughness. In terms of force control effect, when Zhang et al. [19] used a hybrid passive/active force control scheme to grind a tap, the normal grinding force accuracy is under ±5 N. The surface roughness of the machined workpiece in [25] which only employed the robot grinding trajectory planning is 1.189 μm. Zhu et al. [10] combined force control algorithm to grind workpiece, and the values of surface roughness are 0.352 μm and 0.374 μm.

In this paper, a self-adaptive sliding-mode iterative learning method for robotic belt grinding constant-force control is proposed. It not only can effectively compensate for the error caused by the uncertainty of robotic belt grinding, but also has flexible parameter settings and is suitable for actual grinding. Additionally, a simplified force-mapping relationship is proposed. Only a one-dimensional force sensor is required instead of a multi-dimensional force sensor to achieve the control requirements, thereby reducing the complexity of the control system and the cost of the experiment. Firstly, by analysing the contact force between the workpiece and the abrasive belt wheel, the mapping relationship between the grinding force and the force received by sensor is established. Then, the relationship between the grinding normal and tangential force is analyzed and proven by preliminary experiments, and the above mapping relationship is simplified by treating the ratio of the two forces as a constant. The relationship between the grinding deformation and the grinding depth is discussed, and a dynamic model of the robotic belt grinding is established. Then, a force control model based on an adaptive sliding-mode with iterative learning is proposed, and an adaptive control law is designed based on the force feedback error. Finally, the control algorithm proposed in this paper is used to grind angle steel to verify the feasibility of algorithm. The effectiveness of this approach and the feasibility of the simplified force-mapping relationship are further proven by a curved-surface workpiece grinding experiment. 

## 2. Abrasive Belt Grinding Force Analysis

**Figure 1:**
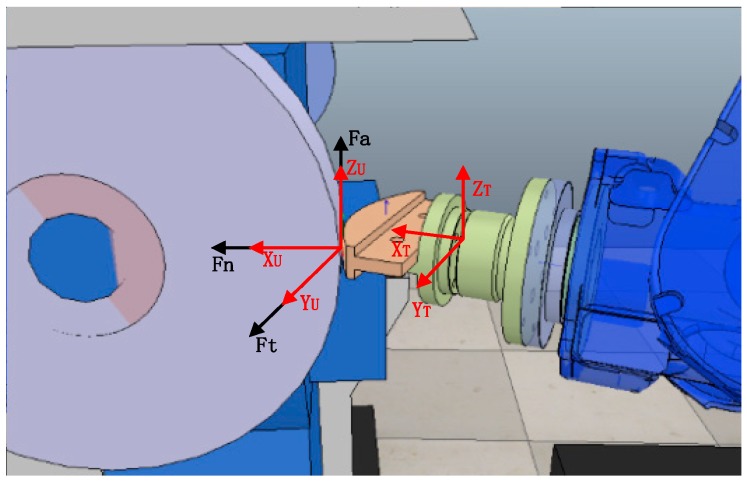
Grinding force and position-pose information of each coordinate system.

During the grinding process, a robot terminal fixture holds a workpiece and grinds it on a belt sander. When the workpiece contacts with the abrasive belt wheel, a certain pressure will be produced on the abrasive belt, and the abrasive grains on the belt will be pressed, which is accompanied by a small plastic deformation, thereby causing the grinding contact force. Figure 1 shows the contact force generated during grinding, and the position-pose relationship between the sensor coordinate system {T} and the belt wheel coordinate system {U}. The contact force can be divided into the normal force *F_n_* perpendicular to the contact surface between the belt wheel and the workpiece, the tangential force $F_{t}$ parallel to the velocity direction of the workpiece and the axial contact force $F_{a}$, which are parallel to the X axis, Y axis and Z axis of the belt wheel coordinate system {U}, respectively. Under normal circumstances, the axial contact force $F_{a}$ is relatively small, so it is ignored. 

**Figure 2:**
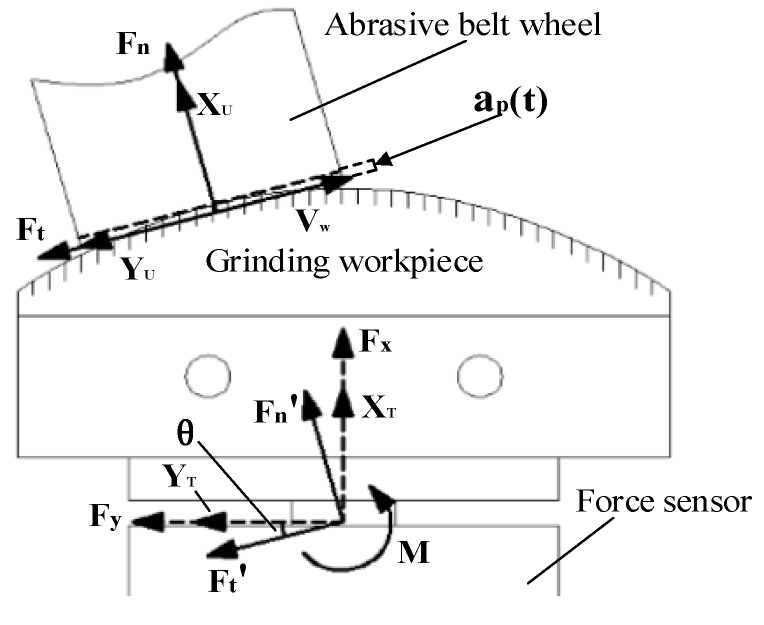
Analysis of the grinding force.

Since the initial grinding trajectory is planned by offline programming [26], it can be ensured that the Z axis of the sensor coordinate system is substantially parallel to the Z axis of the belt wheel coordinate system, so the force analysis can be carried out in the XY plane of the sensor coordinate system {T} or the belt wheel coordinate system {U}. Figure 2 (a top view of Figure 1), shows a force analysis diagram of the abrasive belt grinding process.

The force mapping relation from Figure 2 is:

(1) $$\left\{ \begin{array}{l} \left[\begin{array}{c} F_{t}^{\prime }\\ F_{n}^{\prime } \end{array}\right]=\left[\begin{array}{c} F_{t}\\ F_{n} \end{array}\right]\\ \left[\begin{array}{c} F_{x}\\ F_{y} \end{array}\right]=\left[\begin{array}{cc} -sin\theta & cos\theta \\ cos\theta & sin\theta \end{array}\right]\left[\begin{array}{c} F_{t}^{\prime }\\ F_{n}^{\prime } \end{array}\right] \end{array}\right.$$

where *F_t_* and *F_n_* are the tangential and normal forces on the belt coordinate system, respectively; $F_{t}^{\prime }$ and $F_{n}^{\prime }$ are the forces that transfer $F_{t}$ and $F_{n}$ to the sensor coordinate system, respectively; $F_{x}$ and $F_{y}$ are the forces in the X-axis and Y-axis directions of the sensor, respectively; θ is the angle between the Y-axis direction of the sensor coordinate system {T} and the Y-axis direction of the belt wheel coordinate system {U}. From the above equations, the following equations can be obtained:

(2) $$\left\{ \begin{array}{l} F_{t}=-F_{x}sin\theta +F_{y}cos\theta \\ F_{n}=F_{x}cos\theta +F_{y}sin\theta \end{array}\right.$$

**Figure 3:**
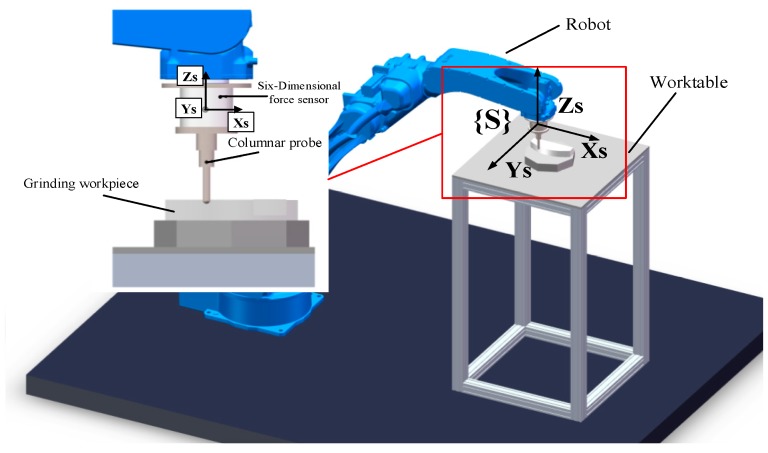
The installation position of the workpiece during tracking.

**Figure 4:**
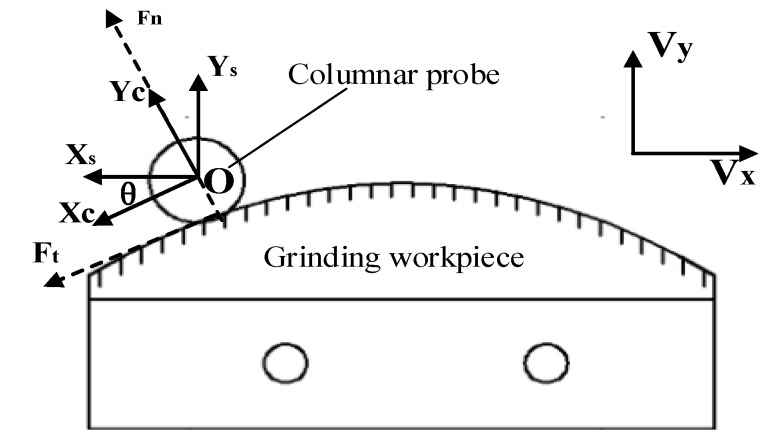
The force analysis diagram for tracking.

Equation (2) shows that when calculating the grinding contact force in the actual grinding process, it is not only necessary to know the force on the sensor, but also need to obtain the angle θ. However, since the position and pose information of the robot and the force information on the sensor cannot be collected synchronously, the position and pose information of the robot cannot be used to obtain the angle θ in the grinding control process, so it is necessary to estimate the angle in advance. The angle θ is estimated by using a method similar to that in reference [27]. The method is to install a columnar probe at the end of the robot and fix the workpiece on the worktable. The installation position of the workpiece is shown in Figure 3 and the force analysis diagram for tracking is shown in Figure 4. 

Where $F_{t}$ and $F_{n}$ are the tangential and normal forces between the probe and the surface; {S} is the coordinate system of sensor; {C} is the coordinate system of surface; $V_{y}$ and $V_{x}$ are the velocities of the robot in the X and Y directions, respectively.

Then the robot probe is made to track the force along the surface of the workpiece, and the angle estimation $\hat{\theta }$ between each position of the workpiece is calculated according to the following equation [27]:

(3) $$\hat{\theta }=\mathrm{atan}(\frac{V_{x}\Delta t}{\Delta y})$$

where Δt is the motion time of the robot in each cycle along the X direction; Δy the offset of the robot in the Y direction.

Finally, we replace the actual angle θ with the estimated value $\hat{\theta }$ and the results show that the average error of the angle is less than 5° in the experiment, which meets the needs of practical application. 

The robot belt grinding schemes in the references are all based on six-dimensional force sensors. Although this method can accurately calculate the normal force in the grinding process, it requires an expensive force sensor and increases the complexity of the control. Therefore, this paper simplifies the force situation by regarding the ratio of the normal force to the tangential force as a constant which acquired through experiments in the case of a particular force range and specific material. The relationship between the tangential force and normal force is as shown in Equation (4):

(4) $$F_{n}=\eta F_{t}$$

Combining Equation (4) with Equation (2), the mapping relation between the force on a one-dimensional sensor and the grinding normal force can be derived as:

(5) $$F_{x}=\frac{cos\theta -sin\theta }{\eta }F_{n}$$

**Figure 5:**
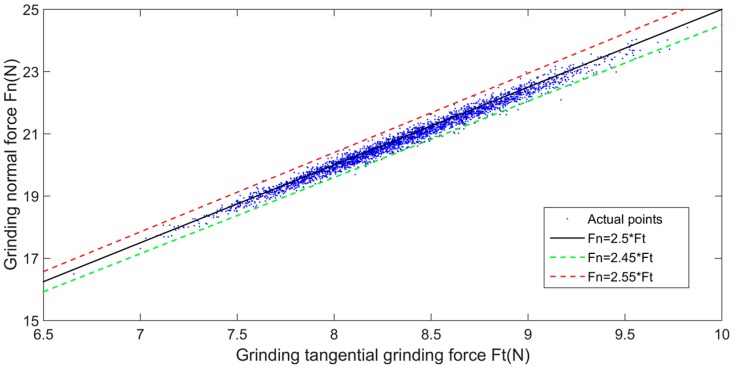
The relationship between normal force and tangential force during grinding.

**Figure 6:**
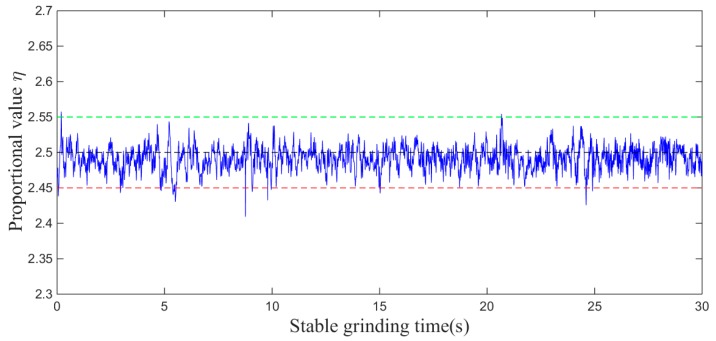
Variation of the proportional value during grinding.

For proving the above conclusion, we design an angle steel grinding experiment with a six-dimensional force sensor, in which the angle steel material is Q235 (S352JR-1.0038, DIN EN 10025-2) and the force $F_{n}$ is controlled at about 20 ± 5 N. Because the angle θ is always kept at zero during the plane grinding process, the tangential and normal forces can be directly expressed by the force received on the six-dimensional sensor. Then, the proportional value η in Equation (4) can be expressed as $F_{x}/F_{y}$. The feasibility of the above Equation (4) can be verified by a plane grinding experiment based on a six-dimensional sensor. Through the experiments, the corresponding normal force and tangential force are shown in Figure 5 and the variation of the proportional value η is shown in Figure 6.

During the grinding process, the value of η essentially fluctuates within 2.5 ± 0.05, with an error of approximately 2%. Therefore, by taking η as 2.5 and substituting it into Equation (5), the relationship between the grinding normal force and the force on the one-dimensional sensor can be obtained as:

(6) $$F_{n}=\frac{F_{x}}{cos\theta -0.4sin\theta }$$

This result shows that it is feasible to use a one-dimensional force sensor instead of a six-dimensional force sensor to calculate the grinding normal force for the control.

## 3. Grinding Dynamics Model

The robot grinding dynamics model is the basis for the robot grinding force control. The traditional grinding dynamics model is written as [28]:

(7) $$f_{p}\left(t\right)=m\ddot{x}\left(t\right)+c\dot{x}\left(t\right)+kx\left(t\right)$$

where $f_{p}\left(t\right)$ is the grinding force; m is the mass coefficient of system; c is the system damping coefficient; k is the process stiffness and these three coefficients are only for later proof, so it is not necessary to know their specific values; x(t) is the position of the machining tool and $\dot{x}\left(t\right)$, $\ddot{x}\left(t\right)$ are its first-order and second-order derivatives, respectively.

However, during the grinding process, in addition to the grinding force $f_{p}\left(t\right)$, the robot is also affected by the deformation force $f_{s}\left(t\right)$ and the oscillating force $f_{q}\left(t\right)$ caused by the uncertainties of the interaction surface [29]. Therefore, the end force f(t) of the robot can be regarded as the sum of these three forces:

(8) $$f\left(t\right)=f_{p}\left(t\right)+f_{s}\left(t\right)+f_{q}\left(t\right)$$

Since the oscillating force $f_{q}\left(t\right)$ in Equation (8) is much smaller than the other two forces, it is instantaneous, unsustainable, unpredictable, and can considered as a disturbance and will not be discussed in this paper. This item is ignored, so Equation (8) can be written as:

(9) $$f\left(t\right)=f_{p}\left(t\right)+f_{s}\left(t\right)$$

Substituting Equation (9) into Equation (7), the robot belt grinding model can be obtained as:

(10) $$f\left(t\right)=m\ddot{x}\left(t\right)+c\dot{x}\left(t\right)+kx\left(t\right)+f_{s}\left(t\right)$$

where f(t) is the force at the end of the robot and $f_{s}\left(t\right)$ is the deformation force of the robot.

To study the relationship between the grinding normal force and grinding depth, the grinding depth $a_{p}\left(t\right)$ can be taken as the research object, and Equation (10) can then be written as:

(11) $$f_{x}\left(t\right)=m{\ddot{a}}_{p}\left(t\right)+c{\dot{a}}_{p}\left(t\right)+ka_{p}\left(t\right)+f_{sx}\left(t\right)$$

where $f_{x}\left(t\right)$ is the grinding force perpendicular to the workpiece, which is shown as the abrasive belt grinding normal force $F_{n}$ in Figure 2; $a_{p}\left(t\right)$ is the grinding depth, which can be equal to x(t); x(t) is the tool position perpendicular to the workpiece surface; and $f_{sx}\left(t\right)$ is the deformation force perpendicular to the workpiece surface.

**Figure 7:**
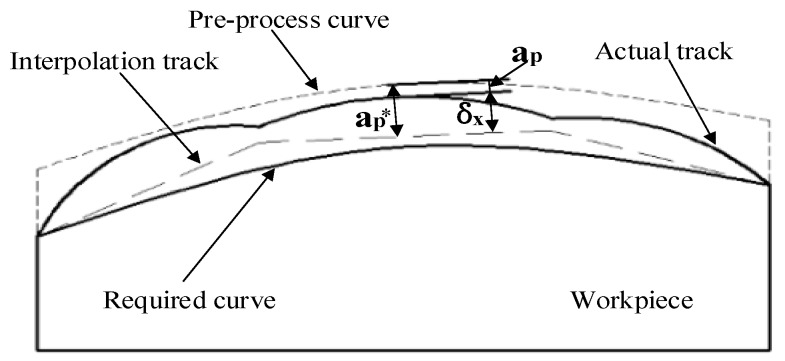
Deviation between planned path and actual path.

During the grinding process, when the robot touches the belt with a feed speed $V_{w}$, the grinding tool extrudes the workpiece and grinds it. At this time, due to the poor rigidity, the robot will be deflected by the reaction force of the grinding, so that it cannot reach the planned depth $a_{p}^{*}$, resulting in part of the workpiece area $\delta_{x}$ not being completely processed. Eventually, there will be an offset between the actual trajectory and the planned trajectory. In the process of the curve trajectory interpolation, the situation shown in Figure 7 will be produced.

According to the above analysis, the deformation $\delta_{x}\left(t\right)$ and deformation force $f_{sx}\left(t\right)$ can be expressed as:

(12) $$\delta_{x}\left(t\right)=a_{p}^{*}\left(t\right)-a_{p}\left(t\right)$$

(13) $$f_{sx}\left(t\right)=k_{s}\delta_{x}\left(t\right)$$

where $a_{p}^{*}\left(t\right)$ is the planned grinding depth and $k_{s}$ is the mechanical arm stiffness of the robot.

Substituting Equation (13) into Equation (11), the deformation-based robot belt sanding dynamics model can be obtained as:

(14) $$f_{x}\left(t\right)=m{\ddot{a}}_{p}\left(t\right)+c{\dot{a}}_{p}\left(t\right)+ka_{p}\left(t\right)+k_{s}\delta_{x}\left(t\right)$$

## 4. Adaptive Sliding-mode Iterative Force Control

This paper chooses the adaptive iterative learning control method because it is simple and accurate and can approximate the target state with arbitrary precision. However, its stability needs to be improved. Therefore, the sliding-mode variable-structure control method is introduced to improve the stability and robustness of the control system and suppress the influence of external disturbances on the robotic belt grinding system. Ultimately, effective control of abrasive belt grinding force is achieved.

### 4.1. Design of Control Law

Before designing the force controller, the appropriate control inputs and outputs should be selected according to the relationship between the parameters in the above-mentioned robotic belt grinding dynamics model. The offset perpendicular to the tangential direction of the workpiece and the surface of the belt is taken as the control object, and the normal force is controlled by adjusting this offset. Since the control target is the desired grinding force, it is necessary to adjust the normal offset according to the error between the desired grinding force and the actual grinding force and the variation speed of this error. Therefore, the relationship can be expressed as:

(15) $$\Delta a_{p}\left(t\right)=Q\left(e_{x}\left(t\right),{\dot{e}}_{x}\left(t\right)\right)$$

(16) $$e_{x}\left(t\right)=f_{x}\left(t\right)-f_{xd}\left(t\right)$$

(17) $${\dot{e}}_{x}\left(t\right)={\dot{f}}_{x}\left(t\right)-{\dot{f}}_{xd}\left(t\right)$$

where $\Delta a_{p}\left(t\right)$ is the normal offset obtained by the control system according to the grinding force error; $e_{x}\left(t\right)$ and ${\dot{e}}_{x}\left(t\right)$ are the grinding force error and its first-order derivative at time *t*, respectively; $f_{x}\left(t\right)$ and ${\dot{f}}_{x}\left(t\right)$ are the actual grinding force and its first-order derivative, respectively; $f_{xd}\left(t\right)$ and ${\dot{f}}_{xd}\left(t\right)$ are the desired grinding force and its first-order derivative, respectively; and Q( ) is determined by the controller and represents the mapping relationship between the grinding force error, its first derivative and the normal offset.

It can be seen from Equations (15) to (17) that the control inputs are the grinding force error and its first derivative, so the sliding surface can be designed as:

(18) $$S_{x}\left(t\right)=\lambda e_{x}\left(t\right)+{\dot{e}}_{x}\left(t\right)$$

where λ is the sliding surface coefficient and $S_{x}\left(t\right)$ is the grinding sliding-mode state.

Substituting Equation (18) into Equation (14) and then combining Equations (16) and (17):

(19) $$S_{xi}=m{\ddot{S}}_{ai}+c{\dot{S}}_{ai}+kS_{ai}+k_{s}S_{\delta i}-S_{f}$$

where *i* is the number of the iteration; $S_{xi}$ is the grinding sliding-mode state at iteration i; $S_{ai}\left(t\right)=\lambda a_{pi}\left(t\right)+{\dot{a}}_{pi}\left(t\right)$ is the grinding depth sliding-mode state; $S_{\delta i}\left(t\right)=\lambda \delta_{xi}\left(t\right)+{\dot{\delta }}_{xi}\left(t\right)$ is the grinding deformation sliding-mode state; and $S_{f}\left(t\right)=\lambda f_{xd}\left(t\right)+{\dot{f}}_{xd}\left(t\right)$ is the desired grinding force sliding-mode state. When $S_{xi}=0$, the grinding sliding-mode state achieves desired state and the system state reaches the control target, and Equation (19) can then be written as:

(20) $$m{\ddot{S}}_{ai}+c{\dot{S}}_{ai}+kS_{ai}=S_{f}-k_{s}S_{\delta i}$$

We can assume that the parameters of the system are unknown and that the system satisfies the following assumptions:

*Assumption 1*: When the workpiece is just in contact with the belt wheel, since the feed depth is set very small, we can assume that the initial grinding depth and the initial state of the system is uniform and repeatable, which is $S_{a1}\left(0\right)=S_{a2}\left(0\right)=\cdots =S_{ai}\left(0\right)$; 

*Assumption 2*: According to the actual situation, the grinding depth $a_{p}$ and the deformation $\delta_{xi}$ are limited, and $f_{xd}$ is a constant force. Some system parameters such as $k_{s}$, c, m, k are bounded as the mass, inertia and stiffness of the whole system are limited during the continuous grinding process. So, based on the above reasons, we can assume that the sliding-mode states $S_{xi}$ and $S_{ai}$, their first derivatives ${\dot{S}}_{xi}$ and ${\dot{S}}_{ai}$, their second derivatives ${\ddot{S}}_{xi}$ and ${\ddot{S}}_{ai}$, and $S_{f}$ are bounded;

According to the Equations (15)–(17), the above assumptions and reference [30], the sliding-mode adaptive iterative control law is designed as:

(21) $$G\left(\Delta a_{p}\left(t\right)\right)=k_{s}S_{\delta i}\left(t\right)$$

(22) $$\Delta a_{p}=k_{p}e_{i}\left(t\right)+k_{d}{\dot{e}}_{i}\left(t\right)+{\hat{\sigma }}_{i}\left(t\right)sgn\left({\dot{e}}_{i}\left(t\right)\right)$$

where:

(23) $$e_{i}\left(t\right)=S_{xd}\left(t\right)-S_{xi}\left(t\right)$$

(24) $${\hat{\sigma }}_{i}\left(t\right)={\hat{\sigma }}_{i-1}\left(t\right)+\gamma {\dot{e}}_{i}\left(t\right)sgn\left({\dot{e}}_{i}\left(t\right)\right)$$

G( ) is the relationship between the normal offset and the sliding surface formed by the deformation of the robot, and is reflected in the actual grinding process; $S_{xd}$ is the desired grinding sliding mode state, whose value is always zero; ${\hat{\sigma }}_{-1}\left(t\right)=0$; γ is the iterative learning rate and *i* is not only the number of iterations but also the number of grinding. If $k_{p}$, $k_{d}$, γ>0 and $Z_{+}$ represents positive integer, $e_{i}\left(t\right)$, ${\dot{e}}_{i}\left(t\right)$ and $\Delta a_{p}\left(t\right)$ are bounded for any positive integer $i\in Z_{+}$ and $lim_{i\to \infty }e_{i}\left(t\right)=lim_{i\to \infty }{\dot{e}}_{i}\left(t\right)=0$, ∀t∈[0,T].

### 4.2. Analysis of Algorithm Stability

To guarantee the stability and convergence of the control system, the Lyapunov method [31] is used to set up the energy function. By analysing the monotonicity and boundedness of the Lyapunov function, the convergence of the error and its first derivative can be proven. According to Assumption 2, we can get the inequality shown in Equation (25), and the Lyapunov function shown in Equation (26) is constructed.

(25) $$\left\{ \begin{array}{l} \left|S_{f}-c{\dot{S}}_{ai}\right|\leq \beta \\ \left|kS_{ai}\right|\geq \varepsilon \end{array}\right.$$

(26) $$W_{i}=V_{i}\left(e_{i}\left(t\right),{\dot{e}}_{i}\left(t\right)\right)+\frac{1}{2}\int_{0}^{t}\gamma^{-1}{\tilde{\sigma }}_{i}^{2}\left(\tau \right)d\tau$$

where β and ε are positive constants, σ is an uncertain item, defined as $\sigma =\beta +Sup_{t\in \left[0,T\right]}\left|S_{xi}\right|-\varepsilon$, and $\tilde{\sigma }\left(t\right)=\sigma -\hat{\sigma }\left(t\right)$. 

According to the previous analysis, $V_{i}\left(e_{i}\left(t\right),{\dot{e}}_{i}\left(t\right)\right)$ can be set as:

(27) $$V_{i}\left(e_{i}\left(t\right),{\dot{e}}_{i}\left(t\right)\right)=\frac{1}{2}\frac{m}{k_{e}}{\dot{e}}_{i}^{2}\left(t\right)+\frac{1}{2}k_{p}e_{i}^{2}\left(t\right)$$

When the robot is in contact with the environment, the environment is often treated as a linear spring [32], and $k_{e}$ in Equation (27) is the environmental stiffness, ie the material stiffness, which can be obtained by consulting the parts material manual. 

To analyze the monotonicity of the Lyapunov function, we set:

(28) $$\Delta W_{i}=W_{i}-W_{i-1}$$

According to Equations (26) and (27), it can obtain:

(29) $$\Delta W_{i}=V_{i}-V_{i-1}+\frac{1}{2}\int_{0}^{t}\gamma^{-1}\left({\tilde{\sigma }}_{i}^{2}\left(\tau \right)+{\tilde{\sigma }}_{i-1}^{2}\left(\tau \right)\right)d\tau$$

The ${\bar{\sigma }}_{i}$ is defined as ${\bar{\sigma }}_{i}={\hat{\sigma }}_{i}-{\hat{\sigma }}_{i-1}$ and substituted into Equation (29):

(30) $$\Delta W_{i}=V_{i}-V_{i-1}-\frac{1}{2}\int_{0}^{t}\gamma^{-1}\left({\bar{\sigma }}_{i}^{2}\left(\tau \right)+2{\tilde{\sigma }}_{i}\left(\tau \right){\bar{\sigma }}_{i}\left(\tau \right)\right)d\tau$$

Integrating the first derivative of $V_{i}\left(e_{i}\left(t\right),{\dot{e}}_{i}\left(t\right)\right)$:

(31) $$V_{i}\left(e_{i}\left(t\right),{\dot{e}}_{i}\left(t\right)\right)=V_{i}\left(e_{i}\left(0\right),{\dot{e}}_{i}\left(0\right)\right)+\int_{0}^{t}\left(\frac{m}{k_{e}}{\dot{e}}_{i}{\ddot{e}}_{i}+k_{p}e_{i}{\dot{e}}_{i}\right)d\tau$$

Since the desired grinding state $S_{xd}$ is always zero and the expected grinding force $f_{xd}$ is a positive constant, ${\ddot{e}}_{i}\left(t\right)={\ddot{S}}_{xd}\left(t\right)-{\ddot{S}}_{xi}\left(t\right)=-{\ddot{S}}_{xi}\left(t\right)=-\lambda {\ddot{f}}_{x}-{\dddot{f}}_{x}$ is obtained. Because the environment was previously treated as a linear spring, $f_{x}=k_{e}a_{p}$, ${\ddot{e}}_{i}\left(t\right)$ can be expressed as ${\ddot{e}}_{i}\left(t\right)=-k_{e}{\ddot{S}}_{ai}\left(t\right)$. Substituting the above equation and into Equation (31),

(32) $$V_{i}\left(e_{i}\left(t\right),{\dot{e}}_{i}\left(t\right)\right)=V_{i}\left(e_{i}\left(0\right),{\dot{e}}_{i}\left(0\right)\right)+\int_{0}^{t}\left(-m{\ddot{S}}_{ai}+k_{p}e_{i}\right){\dot{e}}_{i}d\tau$$

Substituting Equation (19) into Equation (32):

(33) $$V_{i}\left(e_{i}\left(t\right),{\dot{e}}_{i}\left(t\right)\right)=V_{i}\left(e_{i}\left(0\right),{\dot{e}}_{i}\left(0\right)\right)+\int_{0}^{t}\left(S_{xi}-c{\dot{S}}_{ai}-kS_{ai}+S_{f}-G\left(\Delta a_{p}\right)+k_{p}e_{i}\right){\dot{e}}_{i}d\tau$$

Combining inequality (25) and Equation (33), we can get the following inequality:

(34) $$V_{i}\left(e_{i}\left(t\right),{\dot{e}}_{i}\left(t\right)\right)\leq \left\{V_{i}\left(e_{i}\left(0\right),{\dot{e}}_{i}\left(0\right)\right)+\int_{0}^{t}\left(\left(\beta +Sup_{t\in \left[0,T\right]}\left|S_{xi}\right|-\varepsilon \right)sgn\left({\dot{e}}_{i}\right)-G\left(\Delta a_{p}\right)+k_{p}e_{i}\right){\dot{e}}_{i}d\tau \right\}$$

As $\sigma =\beta +Sup_{t\in \left[0,T\right]}\left|S_{xi}\right|-\varepsilon$ and when *t* = 0, $V_{i}\left(e_{i}\left(t\right),{\dot{e}}_{i}\left(t\right)\right)=0$, we can simplify Equation (34) as:

(35) $$V_{i}\left(e_{i}\left(t\right),{\dot{e}}_{i}\left(t\right)\right)\leq \int_{0}^{t}\left(\sigma_{i}sgn\left({\dot{e}}_{i}\right)-G\left(\Delta a_{p}\right)+k_{p}e_{i}\right){\dot{e}}_{i}d\tau$$

Substituting inequality (35) into Equation (30), we can get the following inequality:

(36) $$\Delta W_{i}\leq \int_{0}^{t}\left(\sigma_{i}sgn\left({\dot{e}}_{i}\right)-G\left(\Delta a_{p}\right)+k_{p}e_{i}\right){\dot{e}}_{i}d\tau -V_{i-1}-\frac{1}{2}\int_{0}^{t}\gamma^{-1}\left({\bar{\sigma }}_{i}^{2}\left(\tau \right)+2{\tilde{\sigma }}_{i}\left(\tau \right){\bar{\sigma }}_{i}\left(\tau \right)\right)d\tau$$

Combining Equations (21), (22), (24) and $\tilde{\sigma }\left(t\right)=\sigma -\hat{\sigma }\left(t\right)$, inequality (36) can be simplified as:

(37) $$\Delta W_{i}\leq -V_{i-1}-\frac{1}{2}\int_{0}^{t}\gamma^{-1}\left({\bar{\sigma }}_{i}^{2}+2k_{d}{\ddot{e}}_{i}^{2}\right)d\tau$$

As can be seen from the definition of $V_{i-1}$ in Equation (27), the value of $V_{i-1}$ is always greater than 0. At the same time, it is obvious that $\frac{1}{2}\int_{0}^{t}\gamma^{-1}\left({\bar{\sigma }}_{i}^{2}+2k_{d}{\ddot{e}}_{i}^{2}\right)d\tau$ is not less than 0, so we can obtain the following inequality:

(38) $$\Delta W_{i}\leq \left\{-V_{i-1}-\frac{1}{2}\int_{0}^{t}\gamma^{-1}\left({\bar{\sigma }}_{i}^{2}+2k_{d}{\ddot{e}}_{i}^{2}\right)d\tau \right\}\leq 0$$

It is shown in Equation (38) that $W_{i}$ is a non-incremental sequence. Thus, to prove the boundedness of the entire sequence, it is need to prove that $W_{i}$ has an upper bound.

Deriving $W_{0}$:

(39) $${\dot{W}}_{0}\leq {\dot{e}}_{0}\left({\tilde{\sigma }}_{0}sgn({\dot{e}}_{0})-k_{d}{\dot{e}}_{0}\right)+\frac{1}{2}\gamma^{-1}{\tilde{\sigma }}_{0}^{2}$$

As ${\hat{\sigma }}_{-1}\left(t\right)=0$ and ${\hat{\sigma }}_{i}\left(t\right)={\hat{\sigma }}_{i-1}\left(t\right)+\gamma {\dot{e}}_{i}\left(t\right)sgn\left({\dot{e}}_{i}\left(t\right)\right)$, then:

(40) $${\dot{W}}_{0}\leq -{\dot{e}}_{0}k_{d}{\dot{e}}_{0}+\left({\hat{\sigma }}_{0}+\frac{1}{2}{\tilde{\sigma }}_{0}\right)\gamma^{-1}{\tilde{\sigma }}_{0}$$

Since ${\hat{\sigma }}_{0}\left(t\right)=\sigma \left(t\right)-{\tilde{\sigma }}_{0}\left(t\right)$:

(41) $${\dot{W}}_{0}\leq -{\dot{e}}_{0}k_{d}{\dot{e}}_{0}-\frac{1}{2}{\tilde{\sigma }}_{0}\gamma^{-1}{\tilde{\sigma }}_{0}+\sigma \gamma^{-1}{\tilde{\sigma }}_{0}$$

When α>0:

(42) $$\sigma \gamma^{-1}{\tilde{\sigma }}_{0}\leq \alpha {\left(\gamma^{-1}{\tilde{\sigma }}_{0}\right)}^{2}+\frac{1}{4\alpha }\sigma^{2}$$

Then, Equation (40) can be written as:

(43) $${\dot{W}}_{0}\leq -{\dot{e}}_{0}k_{d}{\dot{e}}_{0}-\frac{1}{2}{\tilde{\sigma }}_{0}\gamma^{-1}{\tilde{\sigma }}_{0}+\alpha {\left(\gamma^{-1}{\tilde{\sigma }}_{0}\right)}^{2}+\frac{1}{4\alpha }\sigma^{2}$$

For the given initial value are bounded, ${\tilde{\sigma }}_{0}$ is bounded. Then:

(44) $$W_{0}\leq \alpha {\left(\gamma^{-1}{\tilde{\sigma }}_{0}{|}_{max}\right)}^{2}+\frac{1}{4\alpha }\sigma_{max}^{2}$$

where $\sigma_{max}=Sup_{t\in [0,T]}\sigma$, ${\tilde{\sigma }}_{0|max}=Sup_{t\in [0,T]}{\tilde{\sigma }}_{0}$.

Therefore, $W_{0}$ is uniformly continuous and bounded on [0,T], and $W_{i}$ is bounded. Furthermore, for any positive integer $i\in Z_{+}$, $e_{i}\left(t\right)$, ${\dot{e}}_{i}\left(t\right)$ and $\Delta a_{p}\left(t\right)$ are bounded.

Then, $W_{i}$ can be rewritten as:

(45) $$W_{i}=W_{0}+\sum_{j=0}^{i}\Delta W_{j}$$

Substituting Equation (45) into Equation (37):

(46) $$W_{i}\leq W_{0}-\sum_{j=0}^{i}\Delta V_{j-1}\leq W_{0}-\frac{1}{2}\sum_{j=0}^{i}\left(k_{p}e_{j-1}^{2}+\frac{m}{k_{e}}{\dot{e}}_{j-1}^{2}\right)$$

(47) $$\sum_{j=0}^{i}\left(k_{p}e_{j-1}^{2}+\frac{m}{k_{e}}{\dot{e}}_{j-1}^{2}\right)\leq 2\left(W_{0}-W_{i}\right)\leq 2W_{0}$$

so it can be obtained that:

(48) $$lim_{i\to \infty }e_{i}\left(t\right)=lim_{i\to \infty }{\dot{e}}_{i}\left(t\right)=0,\;\forall t\in \left[0,T\right]$$

From the above analysis, as the number of iterations increases, the grinding force error will gradually become zero, which means that the actual force will gradually tend to the required force and reflects the Lyapunov stability of the control system.

### 4.3. Design of Control Process

**Figure 8:**
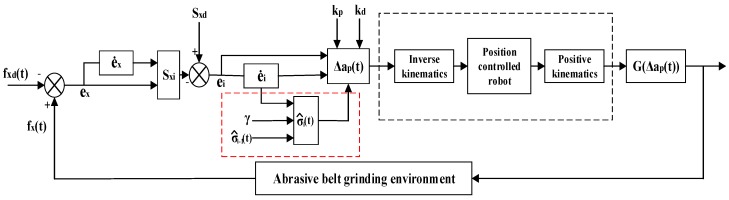
Adaptive sliding-mode iterative control process.

The specific control process is shown in Figure 8.

Where the actual grinding force $f_{x}\left(t\right)$ is collected by the one-dimensional force sensor and transferred into the controller. After a comparison with the desired grinding force $f_{xd}\left(t\right)$, the grinding force error $e_{x}\left(t\right)$ and its first-order derivative ${\dot{e}}_{x}\left(t\right)$ are obtained and used to calculate the actual grinding state $S_{xi}\left(t\right)$. Then, the difference between the actual grinding state $S_{xi}\left(t\right)$ and the desired grinding state $S_{xd}\left(t\right)$ will be determined to obtain the grinding state error $e_{i}\left(t\right)$. With the knowledge of $e_{i}\left(t\right)$ and the adaptive item ${\hat{\sigma }}_{i-1}\left(t\right)$ from the previous iteration, the adaptive item ${\hat{\sigma }}_{i}\left(t\right)$ of the current iteration can be obtained and used to calculate the adjustment of the offset $\Delta a_{p}\left(t\right)$. Finally, the calculated offset $\Delta a_{p}\left(t\right)$ is input to the robot’s control system. And when the portion in the red box in Figure 8 is removed, it is a control structure of non-iterative sliding-mode control.

## 5. Robotic Belt Grinding Experiments

To verify the feasibility of this algorithm, this study separately carried out experiments on grinding angle steel and curved-surface workpieces. Since the angle θ shown in Figure 2 is always zero when grinding angle steel, the force on the one-dimensional sensor can be regarded as the normal grinding force, and the error caused by the simplified force mapping relationship and the angle estimation can also be eliminated. Then, a surface grinding experiment is designed to further verify the effectiveness of the adaptive sliding-mode iteration algorithm and the feasibility of the simplified force-mapping relationship. 

### 5.1. Robotic Belt Grinding System

**Figure 9:**
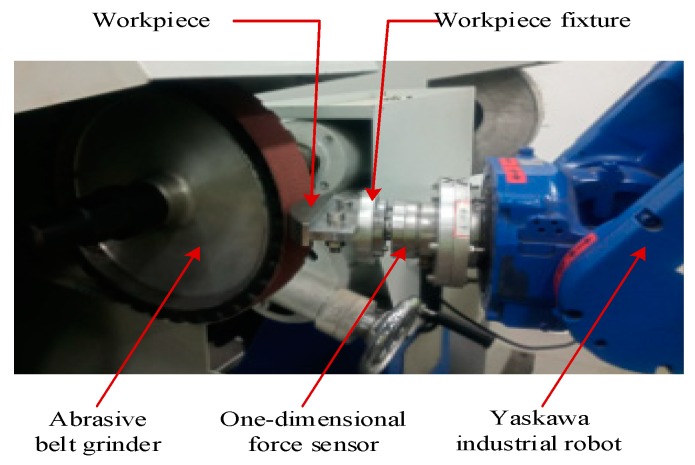
Robot grinding system.

The robotic belt grinding experimental platform is composed of the robot grinding system and robot control system. The main parts of the grinding system, as shown in Figure 9, are a MH24 industrial robot (Yaskawa, Changzhou, China), abrasive belt grinder, workpiece and workpiece fixture, one-dimensional force sensor which is manufactured by Right company (Changzhou, China). The model of the one-dimensional force sensor is a T311, the measuring range is ± 50 N, the actual error is ±0.2 N and it is specially used for robot grinding. The sand belt is a TJ538 type (Little Sun, Foshan, China) and the width of the sand belt is 50 mm, its perimeter is 2100 mm, and its material is zircon corundum. During grinding, the linear velocity of the belt is 8 m/s, the robot linear movement (MOVL) is 25 mm/s. These speed settings are based on processing requirements, including processing materials and technologies.

**Figure 10:**
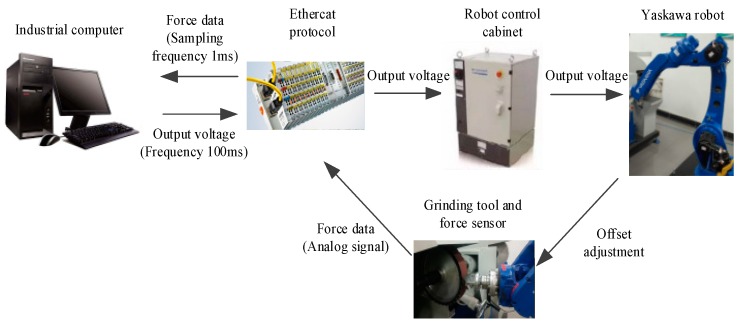
Robot belt grinding control system.

The main parts of the control system, as shown in Figure 10, are the Yaskawa MH24 industrial robot, robot control cabinet, Beckhoff module and industrial computer with TenAsys INtime RTOS from TenAsys (Hillsboro, OR, USA). TenAsys INtime RTOS is a dynamic, deterministic hard real-time operating system for Asymmetric Multi-Processing (AMP) on multi-core x86-compatible processors. INtime RTOS is a full operating system, complete with system services and capabilities that developers expect to see in modern development environments to enable fast and efficient high-performance solutions. 

During the control process, the robot grips the workpiece to take the pre-planned trajectory. The analogue signals collected by the force sensor are then converted into digital signals by a Ethercat interface (Beckhoff fieldbus) module and transmitted to the real-time control system in PC via the Ethercat protocol. The controller uses the control method provided in this paper to calculate the offset and sends it to the Yaskawa robot control cabinet to modify the output pulse and adjust the offset of the robot end-effector. The analogue filter frequency of the force sensor is 2500 Hz, the sampling frequency of the real-time control system is 1 ms, the output voltage of the control system is 100 ms, and the connection between the Ethercat and the Yaskawa robot control cabinet is achieved by the sensor function of the Yaskawa robot.

**Figure 11:**
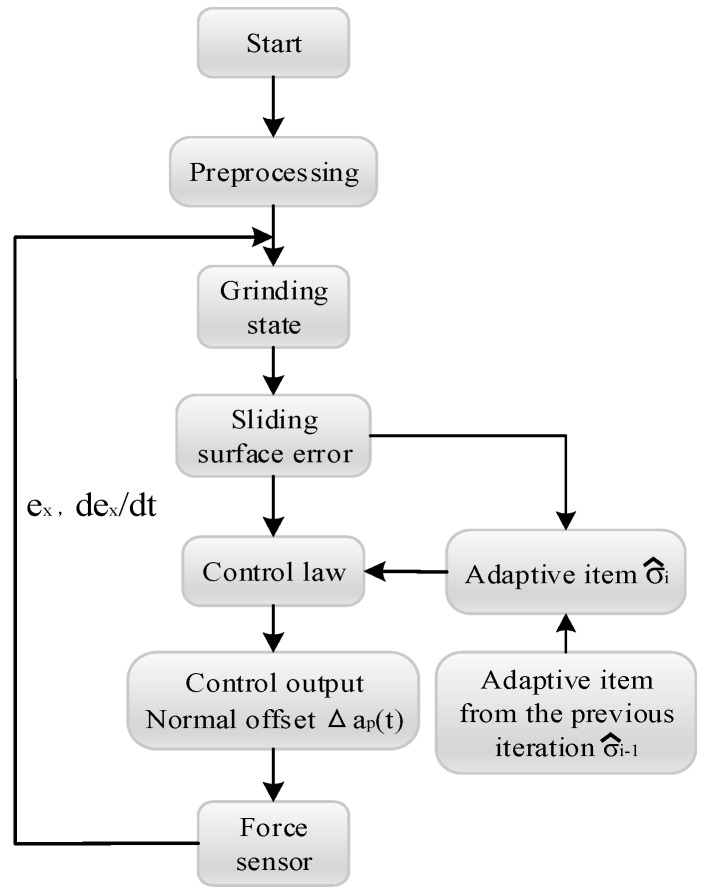
The process of the i-th iteration experiment.

**Figure 12:**
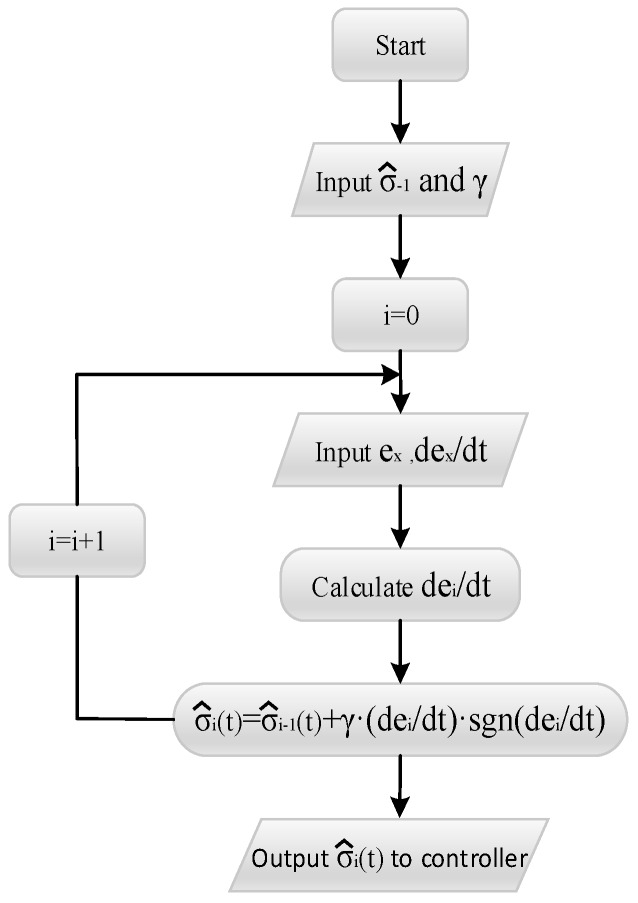
Calculation process of adaptive item.

The process of the *i*-th iteration experiment of the two workpieces is as shown in Figure 11, and the calculation process of the adaptive term ${\hat{\sigma }}_{i}\left(t\right)$ is as shown in Figure 12. It is noteworthy that the gains $k_{p}$, $k_{d}$, λ and γ in the experiment are roughly determined by previous experiments. Different gain values will only affect the convergence speed and iteration times, and the final grinding force will achieve the desired results. 

### 5.2. Angle Steel Grinding Experiment

A Q235 (S352JR-1.0038, DIN EN 10025-2) angle steel sample with a thickness of 3 mm and dimensions of 160 mm × 40 mm is used for the plane grinding test. 

**Figure 13:**
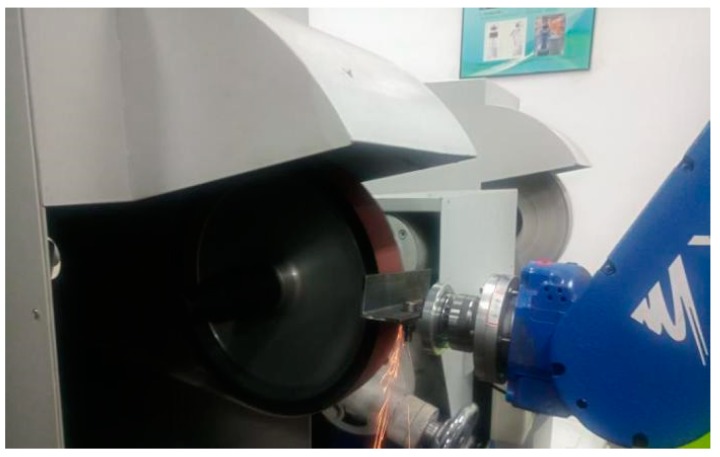
Plane grinding experiment scene.

Before the experiment, the initial grinding trajectory is generated by offline programming, and the offset is then calculated by the force signal feedback from the one-dimensional force sensor in real time and transmitted to the control cabinet to control the robot. The grinding process is shown in Figure 13.

**Figure 14:**
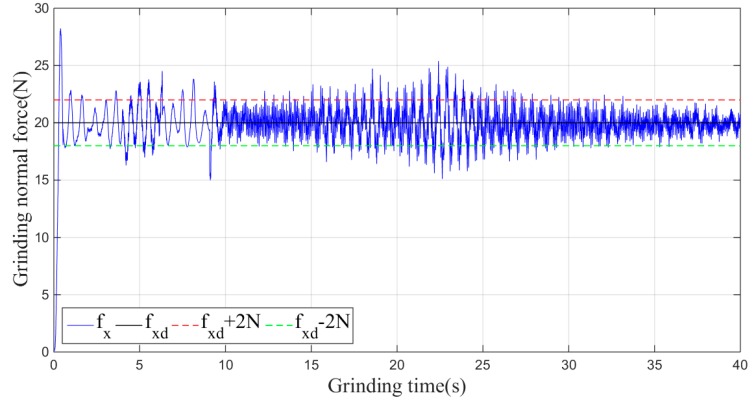
Plane grinding control process without iteration.

In the experiment, the desired grinding force $f_{xd}$ is set to 20 N, and the initial experimental parameters, which are given according to experience, are *k_p_* = 0.055 and *k_d_* = 0.02. The actual grinding force $f_{x}$ under the non-iterative sliding-mode control is shown in Figure 14.

As shown in Figure 14, when the non-iterative sliding-mode control is employed, although the grinding force can be stabilized within a certain range during the grinding process, the convergence speed is slow, and the maximum amplitude of the grinding normal force in the stable machining state fluctuates within 5 N of 20 N.

**Figure 15:**
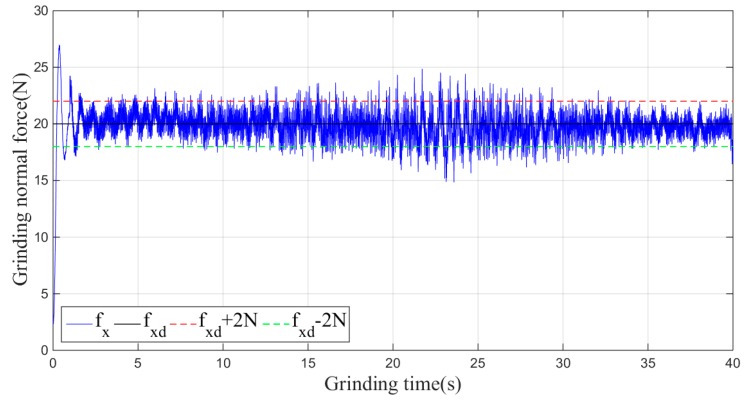
Plane grinding force control process after two iterations.

**Figure 16:**
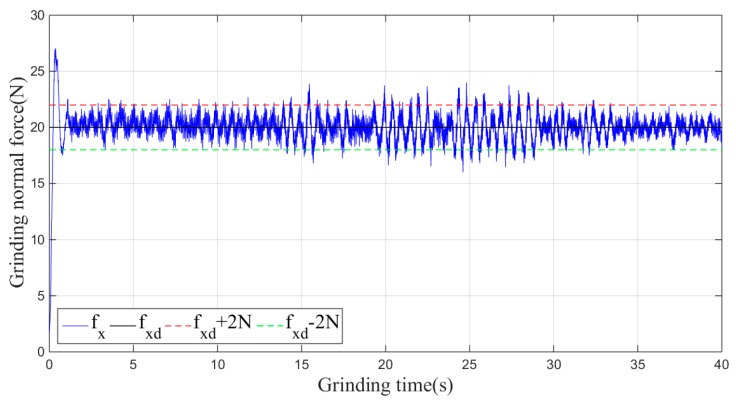
Plane grinding force control process after four iterations.

**Figure 17:**
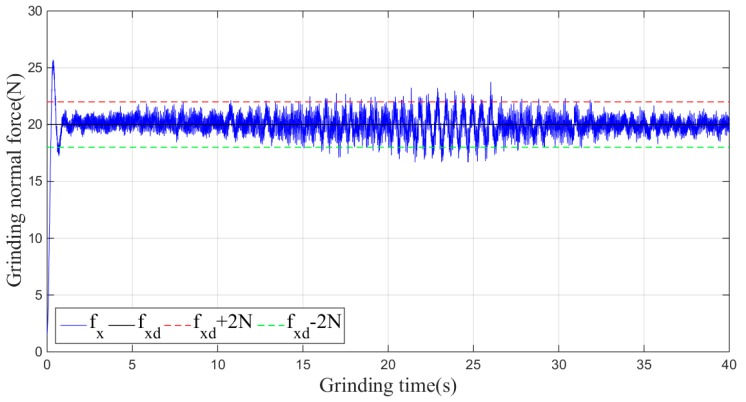
Plane grinding force control process after six iterations.

**Figure 18:**
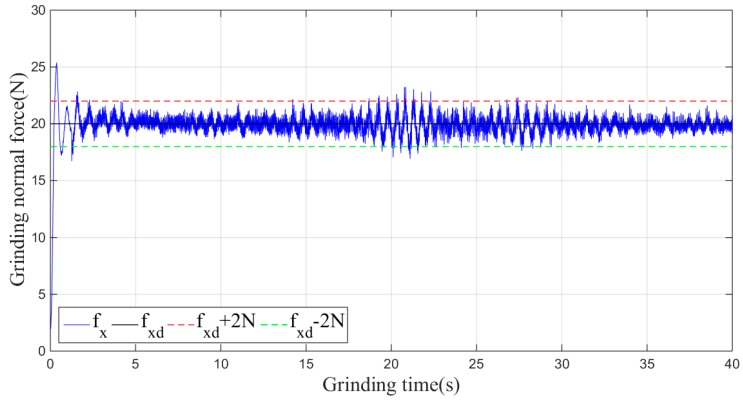
Plane grinding force control process after eight iterations.

**Figure 19:**
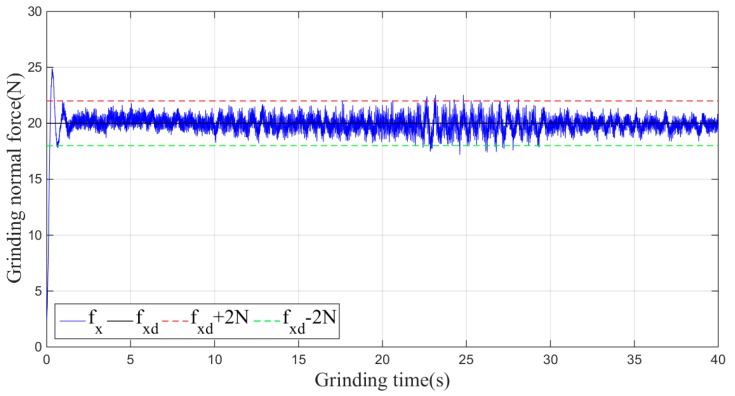
Plane grinding force control process after ten iterations.

Then, the sliding-mode adaptive iterative control method is used for this grinding experiment. The control law shown in Equation (18) is applied. According to experience, the sliding surface coefficient λ is taken as 0.5, and the iteration coefficient γ is taken as 0.3. The grinding normal forces of two, four, six, eight and ten iterations are shown in Figure 15, Figure 16, Figure 17, Figure 18 and Figure 19, respectively.

**Figure 20:**
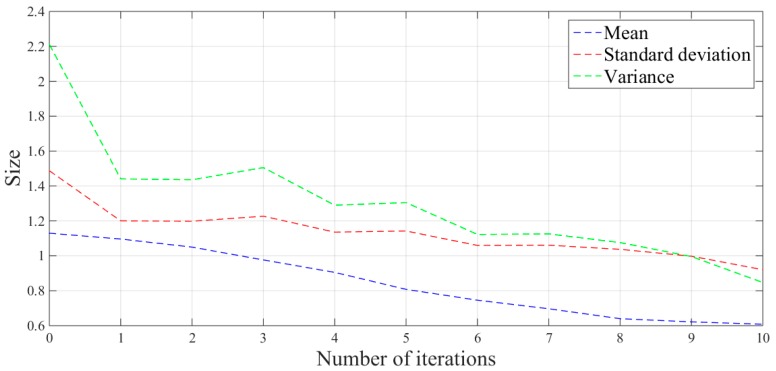
Error analysis picture of plane grinding force.

The mean, standard deviation and variance of the absolute values of the force errors during the iteration are calculated and shown in Table 1 and Figure 20.

From the above series of figures, after ten iterations, the grinding force during the stable grinding state fluctuates around 20 N with amplitude of 2 N. As seen from Table 1 and Figure 20, the mean, standard deviation and variance of the absolute value of the grinding force error decrease with the increasing number of iterations. Compared to the non-iterative sliding-mode control, they are reduced by 46%, 38%, and 62%, respectively. Moreover, with an increase in the number of iterations, the convergence speed is also greatly improved. The results indicate that the adaptive sliding-mode iteration method has better performance in reducing the deviation of the grinding force.

**Figure 21:**
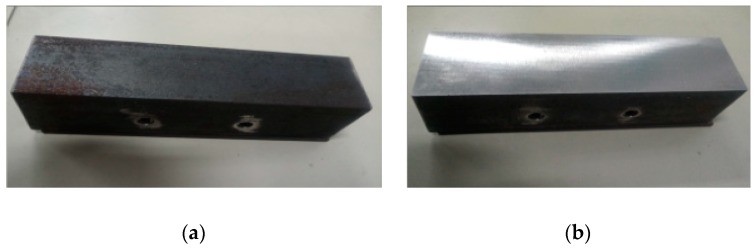
Comparison of angled steel before and after grinding. (**a**) Angle steel before grinding; (**b**) Angle steel after grinding

**Figure 22:**
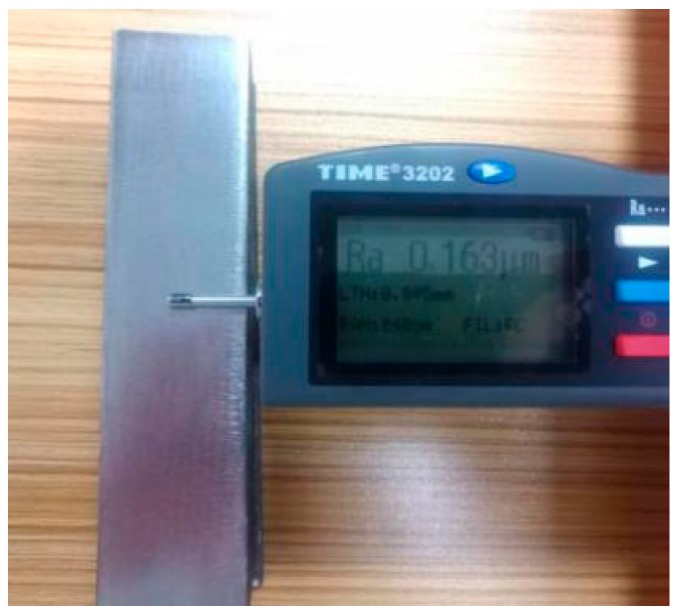
Roughness measurement process of angle steel.

Meanwhile, to judge the processing effect of the robot’s constant-force grinding, it is necessary to detect the surface roughness of the workpiece. Figure 21 shows a comparison of the workpiece before and after grinding, and a roughometer (Figure 22) is then used to measure the roughness of the machined workpiece. The roughometer is manufactured by Beijing TIME High Technology Ltd., (Beijing, China) its model is TIME3202, the range of Ra and Rq is 0.005–16 μm, the range of Rz is 0.02–160 μm, and the indication variability is ≤6%. 

**Figure 23:**
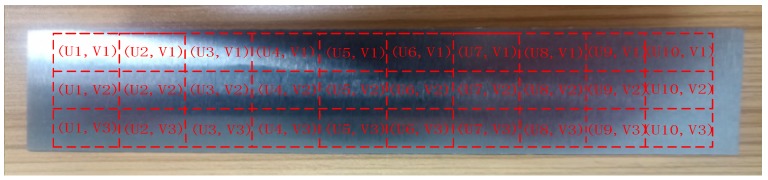
Measurement area division of angle steel.

The specific measurement method is to divide the processed workpiece into areas, as shown in Figure 23, and measure the roughness of each area with a roughometer. The measurement results are recorded in Table 2.

**Figure 24:**
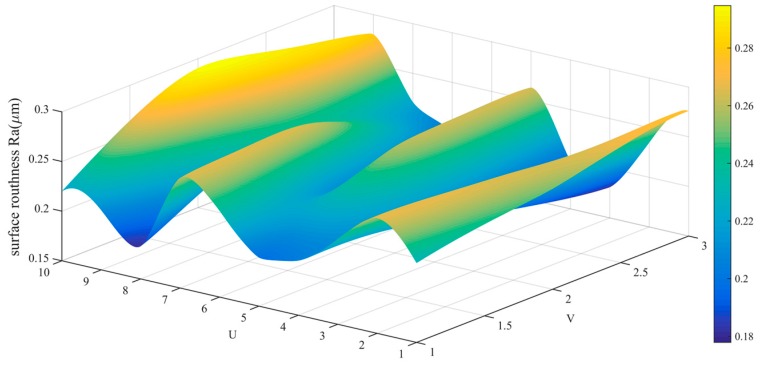
Roughness (Ra) distribution cloud picture of angle steel.

**Figure 25:**
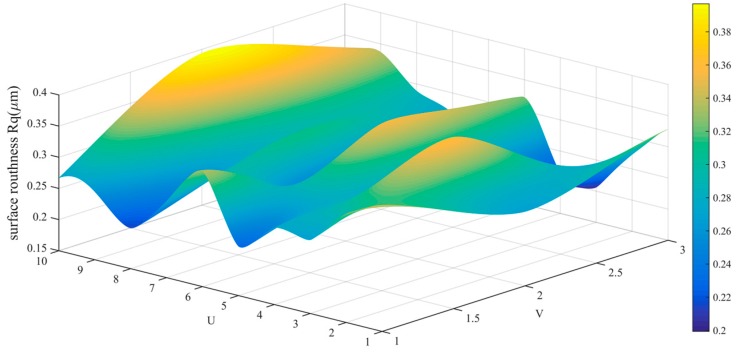
Roughness (Rq) distribution cloud picture of angle steel.

**Figure 26:**
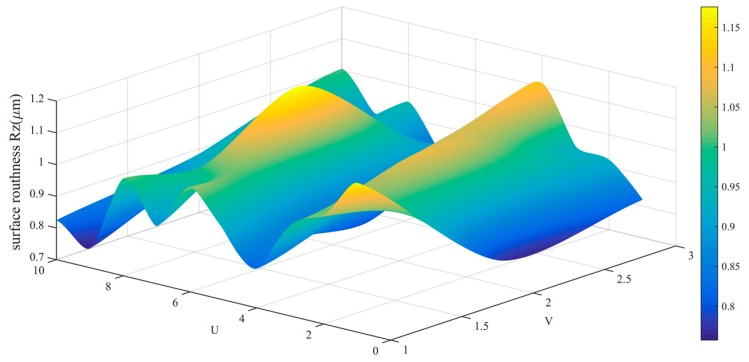
Roughness (Rz) distribution cloud picture of angle steel.

The data in Table 2 is drawn into a roughness cloud picture, as shown in Figure 24, Figure 25 and Figure 26. It can be seen that the surface roughness Ra, Rq and Rz of workpiece are within 0.181–0.290 μm, 0.206–0.394 μm and 0.761–0.171 μm. Their average values and standard deviation are also small and all bigger than the variability of the roughness meter itself.

This result indicates that the surface roughness distribution of the workpiece is uniform, and the effectiveness and stability of the control method is also reflected from the side.

### 5.3. Curved-surface Workpiece Grinding Experiment

**Figure 27:**
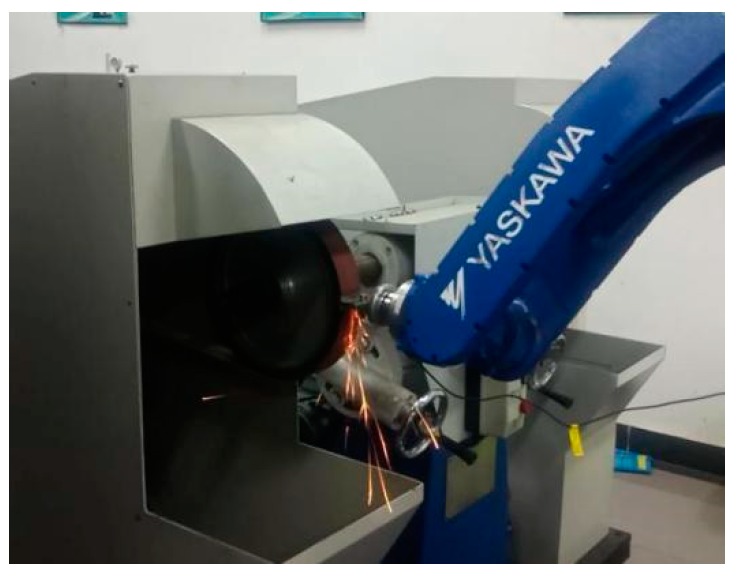
Surface grinding experiment scene.

Curved-surface workpieces made of 45# steel (C45+QT-1.1191, DIN EN 10083-1) and with the contour of a spline curve are used for the grinding experiment. A pretreatment method similar to that used in the plane grinding experiment is used to reduce the influence of uncertain disturbances on the experimental results. The surface grinding process is shown in Figure 27.

**Figure 28:**
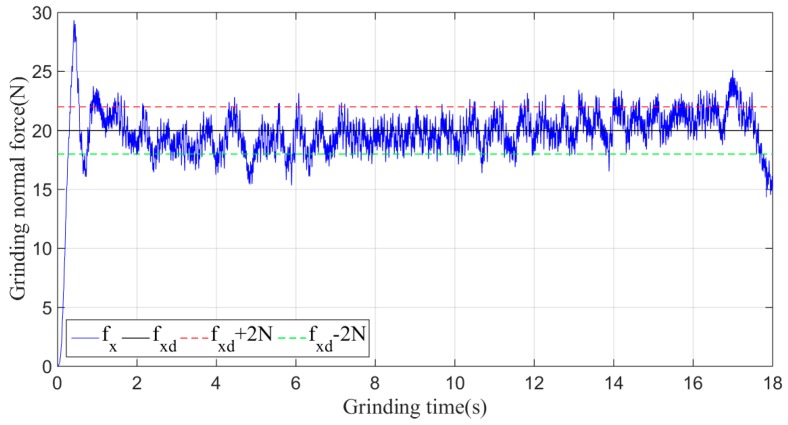
Surface grinding control process without iteration.

In the experiment, the desired grinding force $f_{xd}$ is set to 20 N, and the initial experimental parameters, which are given according to experience, are *k_p_* = 0.04 and *k_d_* = 0.04. Then, the force received on the one-dimensional sensor is converted into the normal force by the relationship shown in Equation (6). Finally, the calculated grinding normal force is controlled, and the actual grinding force $f_{x}$ under the non-iterative sliding-mode control is shown in Figure 28.

Unlike in angle steel grinding, the maximum normal force fluctuation reaches 6 N, and there is a steady-state error when the grinding curved surface is only under sliding-mode control.

**Figure 29:**
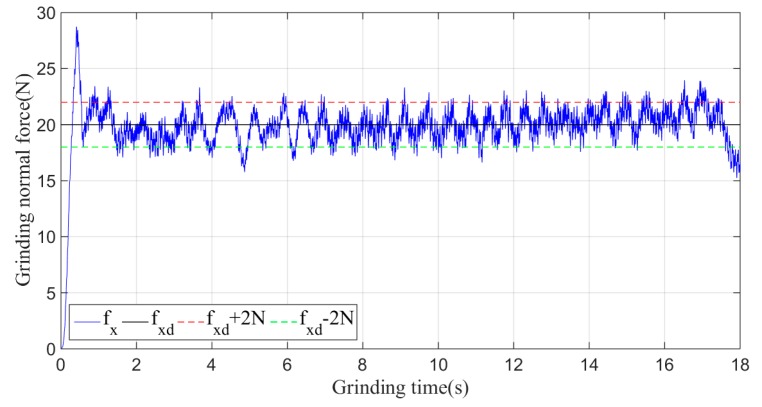
Surface grinding force control process after two iterations.

**Figure 30:**
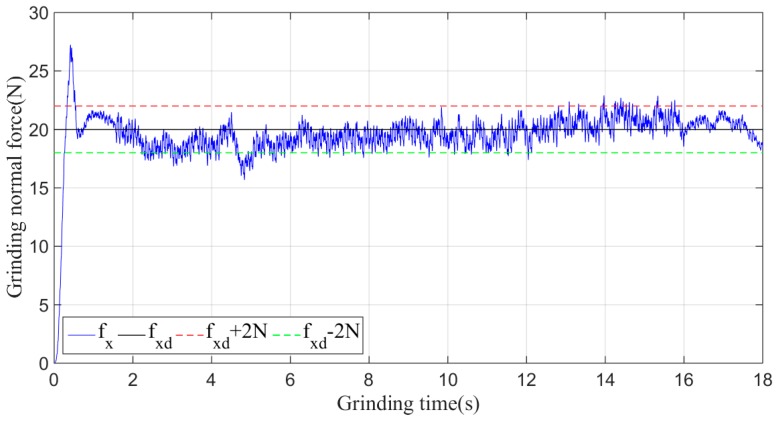
Surface grinding force control process after four iterations.

**Figure 31:**
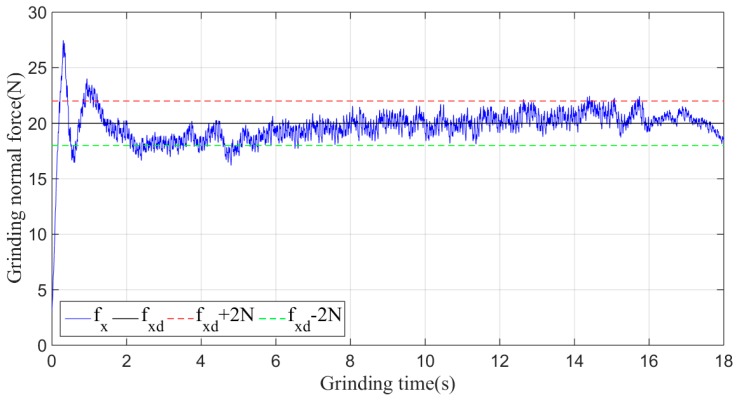
Surface grinding force control process after six iterations.

**Figure 32:**
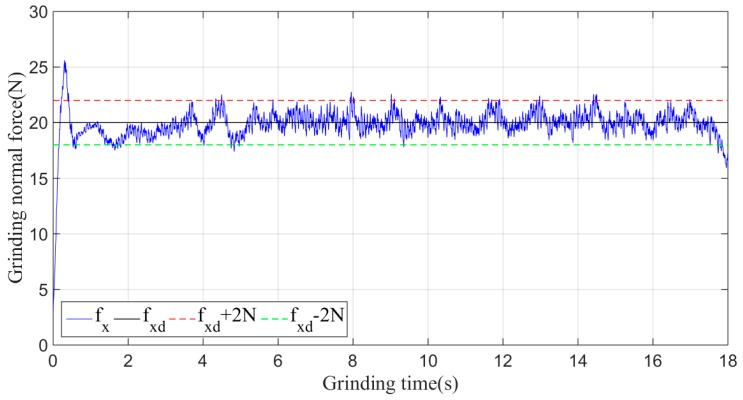
Surface grinding force control process after eight iterations.

**Figure 33:**
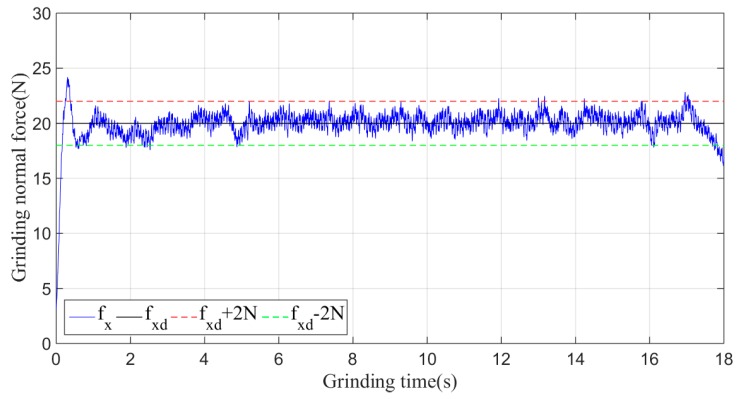
Surface grinding force control process after ten iterations.

Then the sliding-mode adaptive iterative control method is used to control the grinding normal force. The control law shown in Equation (18) is applied, with the sliding surface coefficient λ taken as 0.5 and the iteration coefficient γ taken as 0.4 according to experience. The grinding normal forces of two, four, six, eight and ten iterations are shown in Figure 29, Figure 30, Figure 31, Figure 32 and Figure 33, respectively. 

**Figure 34:**
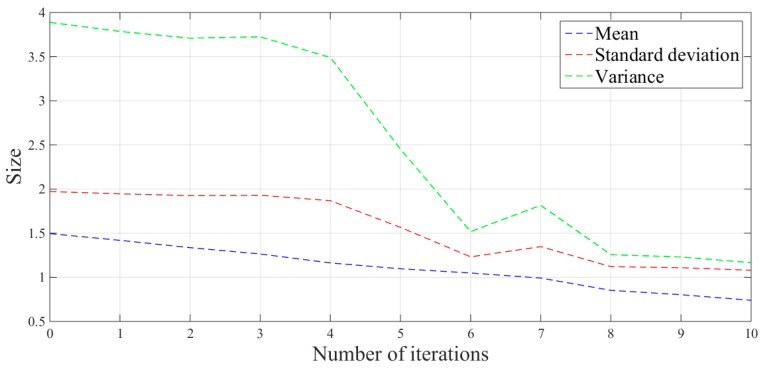
Error analysis picture of surface grinding force.

The mean, standard deviation and variance of the absolute values of the force errors during the iteration are calculated and shown in Table 3 and Figure 34.

Compared with the non-iterative sliding-mode control and each iterative process, the amplitude of the surface grinding force decreases with an increase in the number of iterations, and finally stabilizes at approximately 2 N. Similar to the plane grinding force effect, the mean, standard deviation and variance of the absolute value of the surface grinding force error also showed a downward trends, and decreased by 51%, 45% and 70%, respectively, compared to the values for the non-iterative sliding-mode control. The convergence effect and stability of the grinding force are also significantly improved. 

**Figure 35:**
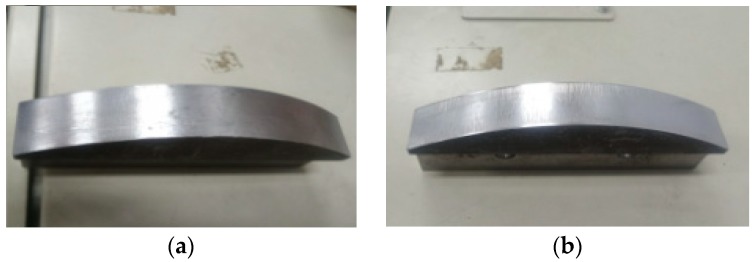
Comparison of the curved-surface workpiece before and after grinding. (**a**) Curved-surface workpiece before grinding; (**b**) Curved-surface workpiece after grinding.

**Figure 36:**
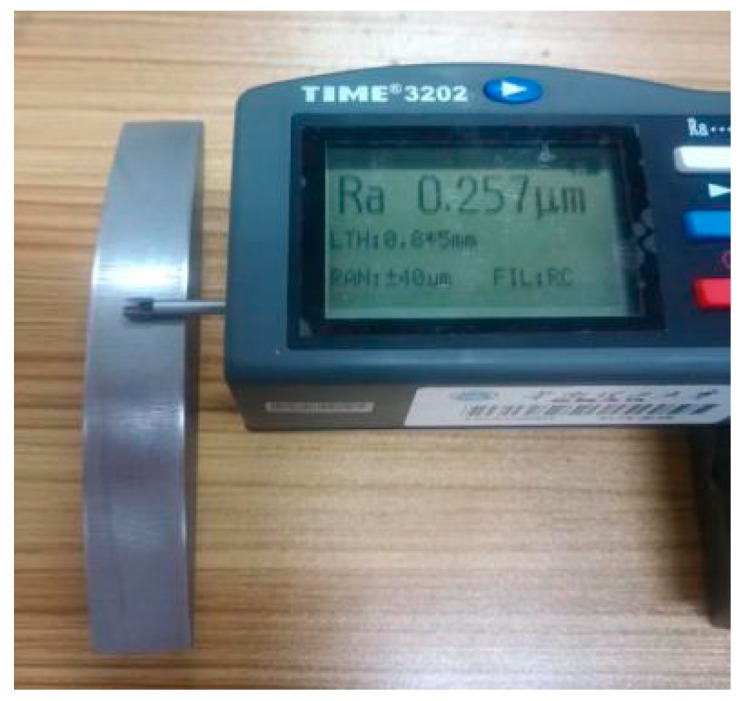
Roughness measurement process of the curved-surface workpiece.

**Figure 37:**
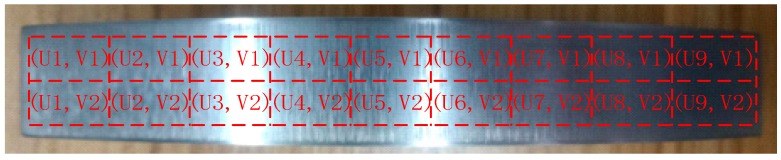
Measurement area division of the curved-surface workpiece.

Similarly, the roughness of the machined surface workpiece was tested, and the curved-surface workpiece before and after grinding is shown in Figure 35. Then, using the same measurement process (Figure 36) and method as in the angle steel grinding experiment, the measurement area of the processed curved-surface is divided as shown in Figure 37.

**Figure 38:**
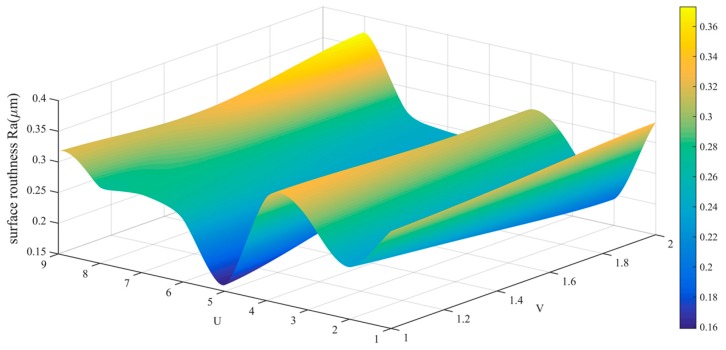
Roughness (Ra) distribution cloud picture of the curved-surface workpiece.

**Figure 39:**
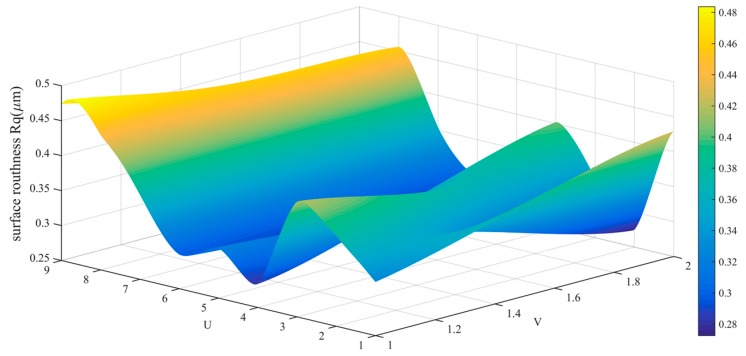
Roughness (Rq) distribution cloud picture of the curved-surface workpiece.

**Figure 40:**
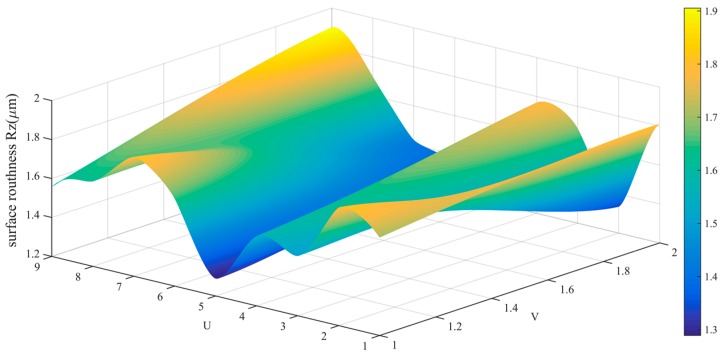
Roughness (Rz) distribution cloud picture of the curved-surface workpiece.

The data in Table 4 is drawn into a roughness cloud picture, as shown in Figure 38, Figure 39 and Figure 40. It can be seen that the surface roughness Ra, Rq and Rz of workpiece are within 0.160–0.373 μm, 0.275–0.474 μm and 1.289–1.901 μm. Their average values and standard deviation are also small and all bigger than the variability of the roughness meter itself. This result indicates that the surface roughness distribution of the workpiece is more uniform and further reflects the effectiveness and stability of the control method.

These experimental results show that the algorithm can achieve effective control effects for both planar and curved workpieces, and the grinding force is quickly stabilized within a certain range, which shows the feasibility and stability of the algorithm. Moreover, it can be concluded from the result of the surface grinding experiment that the control algorithm effectively suppresses the force fluctuation that is caused by the variation of the curvature and the simplified force-mapping relationship. In addition, the force control effect of the proposed algorithm and the surface quality of the machined workpiece in this paper are better than the previous literature. These results also indicate that it is feasible to consider the ratio of the normal force and tangential force as a constant and combined it with the control algorithm.

## 6. Conclusions

In this paper, an adaptive sliding-mode iterative constant-force control method for belt grinding based on a one-dimensional force sensor is proposed. It simplifies the force-mapping relationship, reduces the research cost and reduces the amplitude of the force fluctuation in the process of robotic belt grinding. To map the grinding normal force to the one-dimensional force sensor, the force of the belt grinding is first analyzed, and the ratio of the grinding normal force to the tangential force is regarded as a positive constant, as is proven by a preliminary experiment. For achieving the purpose of control, the relationship between the deformation and grinding depth during grinding was analyzed and a dynamic model was established. Considering the control accuracy, the adaptive iterative learning method is introduced, and the combination of sliding-mode control and iterative learning is used to improve the stability and robustness of the control system. 

The experimental results show that compared with the non-iterative sliding-mode control and each iterative control, the proposed control scheme has the advantages of improving the force convergence speed and reducing the force fluctuation amplitude. The fluctuation amplitude of the grinding force decreased from 5 N or 6 N to 2 N. Meanwhile, with an increase in the number of iterations, the mean, standard deviation and variance of the absolute error generally downward trends, which reflects the effectiveness of the algorithm and the feasibility of the force simplification from the side. The ultimate goal of controlling the constant force is to improve the machining quality of workpieces. Comparing the workpieces before and after grinding, the surface quality of the workpiece is significantly improved, the surface roughness is small and its distribution is uniform. These results indicate that the algorithm proposed in this paper can achieve the ultimate goal. Many current studies on robot surface grinding are based on multi-dimensional force sensors. In this paper, by simplifying the force-mapping relationship, a better force control effect can be achieved using a one-dimensional force sensor, which provides another method for subsequent research.

The adaptive sliding-mode iterative constant-force control method proposed in this paper can achieve better force control effect and has certain flexibility. However, the positioning accuracy of the robot still has some influence on the grinding force and grinding accuracy. Hence, in the future, laser tracker and related algorithms can be proposed to compensate the positioning accuracy of the robot, and then be used to improve the grinding effect with the adaptive sliding-mode iterative constant-force control algorithm.

## Figures and Tables

**Table 1:** Absolute value error analysis of the plane grinding force.

Iteration Number	Mean	Standard Deviation	Variance
0	1.1297	1.4872	2.2118
1	1.0962	1.2004	1.4410
2	1.0500	1.1982	1.4359
3	0.9765	1.2270	1.5055
4	0.9052	1.1358	1.2900
5	0.8080	1.1424	1.3051
6	0.7459	1.0595	1.1225
7	0.6968	1.0610	1.1257
8	0.6399	1.0372	1.0758
9	0.6217	0.9979	0.9958
10	0.6082	0.9205	0.8473

**Table 2:** Roughness of angle steel after grinding (μm).

	V1			V2			V3		
	Ra	Rq	Rz	Ra	Rq	Rz	Ra	Rq	Rz
**U1**	0.230	0.352	1.171	0.251	0.269	0.777	0.276	0.328	0.820
**U2**	0.266	0.325	1.042	0.258	0.287	0.949	0.235	0.273	0.921
**U3**	0.236	0.268	0.986	0.206	0.360	1.078	0.181	0.206	0.938
**U4**	0.205	0.273	0.824	0.221	0.270	0.996	0.198	0.215	1.112
**U5**	0.199	0.229	0.921	0.257	0.353	0.794	0.263	0.322	1.035
**U6**	0.237	0.335	1.031	0.205	0.212	0.921	0.198	0.257	0.894
**U7**	0.261	0.273	0.882	0.261	0.309	1.175	0.201	0.268	0.813
**U8**	0.183	0.215	1.007	0.241	0.289	0.844	0.221	0.289	0.951
**U9**	0.210	0.259	0.761	0.271	0.355	0.953	0.280	0.337	0.889
**U10**	0.220	0.267	0.824	0.290	0.394	0.885	0.227	0.310	1.005
**Mean**	0.225	0.280	0.945	0.246	0.310	0.937	0.228	0.281	0.938
**Standard deviation**	0.027	0.0446	0.125	0.028	0.0551	0.124	0.035	0.0456	0.093

**Table 3:** Absolute value error analysis of the surface grinding force.

Iteration Number	Mean	Standard Deviation	Variance
0	1.4953	1.9718	3.8880
1	1.4192	1.9457	3.7857
2	1.3361	1.9259	3.7091
3	1.2646	1.9298	3.7241
4	1.1632	1.8682	3.4902
5	1.0971	1.5658	2.4517
6	1.0490	1.2320	1.5178
7	0.9918	1.3476	1.8160
8	0.8523	1.1211	1.2569
9	0.8031	1.1092	1.2303
10	0.7398	1.0799	1.1662

**Table 4:** Roughness of the curved-surface workpiece after grinding (μm).

	V1			V2		
	Ra	Rq	Rz	Ra	Rq	Rz
**U1**	0.309	0.327	1.703	0.333	0.429	1.812
**U2**	0.235	0.379	1.806	0.193	0.275	1.335
**U3**	0.299	0.415	1.507	0.258	0.335	1.687
**U4**	0.218	0.284	1.558	0.309	0.402	1.769
**U5**	0.160	0.321	1.289	0.257	0.312	1.519
**U6**	0.252	0.299	1.648	0.242	0.296	1.371
**U7**	0.277	0.371	1.812	0.261	0.354	1.425
**U8**	0.274	0.443	1.636	0.373	0.456	1.699
**U9**	0.317	0.474	1.558	0.240	0.315	1.901
**Mean**	0.271	0.368	1.613	0.274	0.353	1.613
**Standard deviation**	0.051	0.066	0.161	0.055	0.063	0.206
